# Efficacy and Safety of Anthocyanin-Rich Extract in Patients with Ulcerative Colitis: A Randomized Controlled Trial

**DOI:** 10.3390/nu16234197

**Published:** 2024-12-04

**Authors:** Luc Biedermann, Michael Doulberis, Philipp Schreiner, Ole Haagen Nielsen, Frans Olivier The, Stephan Brand, Sabine Burk, Petr Hruz, Pascal Juillerat, Claudia Krieger-Grübel, Kristin Leu, Gabriel E. Leventhal, Benjamin Misselwitz, Sylvie Scharl, Alain Schoepfer, Frank Seibold, Hans Herfarth, Gerhard Rogler

**Affiliations:** 1 Department of Gastroenterology and Hepatology, University Hospital Zurich, University of Zurich, 8091 Zurich, Switzerland; luc.biedermann@usz.ch (L.B.); doulberis@gmail.com (M.D.); philippschreiner@hotmail.com (P.S.); fransolivier.the@triemli.zuerich.ch (F.O.T.); sabine.burk@usz.ch (S.B.); leu.kristin@gmail.com (K.L.); sylvie.scharl@usz.ch (S.S.); 2 Gastroklinik, Private Gastroenterological Practice, 8810 Horgen, Switzerland; 3 Division of Gastroenterology and Hepatology, Medical University Department, Kantonsspital Aarau, 5001 Aarau, Switzerland; 4 Department of Gastroenterology, Herlev and Gentofte Hospital, University of Copenhagen, 2730 Herlev, Denmark; ole.haagen.nielsen@regionh.dk; 5 Department of Gastroenterology and Hepatology, Kantonsspital St. Gallen, 9007 St. Gallen, Switzerland; stephan.brand@kssg.ch (S.B.); claudia.krieger-gruebel@kssg.ch (C.K.-G.); 6 Department of Gastroenterology, Clarunis-University Center for Gastrointestinal and Liver Diseases, 4052 Basel, Switzerland; petr.hruz@clarunis.ch; 7 Intesto Crohn and Colitis Center, 3012 Bern, Switzerland; juilleratp@intesto.ch (P.J.); seiboldf@intesto.ch (F.S.); 8 Department of Visceral Surgery and Medicine, Inselspital Bern University Hospital, University of Bern, 3010 Bern, Switzerland; benjamin.misselwitz@insel.ch; 9 Department of Gastroenterology and Hepatology, Centre Hospitalier Universitaire Vaudois, University of Lausanne, 1011 Lausanne, Switzerland; alain.schoepfer@chuv.ch; 10 Division of Gastroenterology and Hepatology, University of North Carolina at Chapel Hill, Chapel Hill, NC 27514, USA; hans_herfarth@med.unc.edu

**Keywords:** ulcerative colitis, anthocyanin-rich extract (ACRE), inflammatory bowel disease (IBD), complementary therapy, bilberries

## Abstract

**Background:** Bilberries are effective in inducing clinical, endoscopic, and biochemical improvement in ulcerative colitis (UC) patients. The aim of this study was to investigate the efficacy of anthocyanin-rich extract (ACRE), the bioactive ingredient of bilberries, in a controlled clinical trial in moderate-to-severe UC. **Methods:** A multi-center, randomized, placebo-controlled, double-blind study with a parallel group was conducted. Initially, the study was planned for 100 patients; nevertheless, it prematurely ended due to COVID-19. Patients had moderate-to-severe active UC at screening (a Mayo score of 6–12, an endoscopic sub-score ≥ 2) and were randomized at baseline. The primary endpoint was a clinical response (week 8, a total Mayo score reduction ≥ 3 points). Fecal calprotectin (FC) and a centrally read endoscopic response were among the secondary endpoints. **Results:** Out of 48 patients (6 Swiss centers), 34 were randomized. Eighteen ACRE and eight placebo patients could be analyzed (per protocol set). Half (9/18) of ACRE patients and 3/8 of placebo patients responded clinically (*p* = 0.278). An improvement in the Mayo score was observed in the ACRE arm (77.8% vs. 62.5% placebo). FC dropped from 1049 ± 1139 to 557 ± 756 μg/g for ACRE but not for the placebo group (947 ± 1039 to 1040 ± 1179; *p* = 0.035). Serious adverse events were rare. **Conclusions:** ACRE treatment did not yield significant superiority to the placebo. Furthermore, the placebo response was unusually high. Moreover, there was a significant calprotectin decrease at the end of treatment, indicative of ACRE efficacy in UC.

## 1. Introduction

Ulcerative colitis (UC) and Crohn’s disease represent the two main types of inflammatory bowel diseases (IBDs). UC is an idiopathic, chronic, relapsing pathology of the colon affecting mainly young adults with a peak age range between 30 and 40 years with no gender predominance. The most typical symptoms include severe, often bloody diarrhea and abdominal discomfort. Despite the variability in the clinical course, most patients suffer from recurrent flares and an unpredictable disease course [1,2]. Quality of life may be severely impaired by persistent, frequent, recurrent, or waxing and waning symptoms [3].

Although UC is incurable, approximately two-thirds of patients with moderate disease activity can be successfully treated with mesalamine (5-aminosalicylic acid, 5-ASA) [4,5]. Nevertheless, patients who do not achieve a sufficient response remain a clinical challenge, and up to 10% of all patients have to undergo a colectomy in the long term, according to a recent (2023) systematic review and meta-analysis [6].

While there is a plethora of potentially effective drugs to treat UC, the overall profile of those is far from being ideal. They harbor a considerable risk of short- and long-term toxicity and numerous side effects [7,8,9]. Moreover, the annual costs of newer treatment options (such as biologics and small molecules) are often high [10,11]. Most importantly, even the latest treatments demonstrate efficiency in only a proportion of UC patients (“therapeutic ceiling”) [12,13]. Therefore, the development of further medical treatment options, with favorable cost–benefit ratios and salutary side-effect profiles, clearly represents an urgent requirement for UC management. In this respect, herbal and natural compound treatments constitute an appealing therapy option for UC patients. Additionally, there is growing demand among Swiss patients for natural therapy options.

In this context, several relevant studies report generally positive results regarding natural compounds such as curcuma [14] (*Curcuma zedoaria*, an Indian spice), Qing-Dai (*Indigo naturalis*) [15], or boswellia tree resin (*Boswellia serrata*), as well as aloe vera [16,17] on UC patients. In this respect, a relevant systematic review and meta-analysis on the same topic was shortly published in *Nutrients*. It was deduced that at least certain herbal remedies are promising with positive outcomes, and further evidence is, therefore, warranted [15].

Interest in natural flavonoids, a subgroup of polyphenols, has been increasing in recent years due to their beneficial effects on many conditions, including cardiovascular disease and cancer. Anthocyanins (ACs), compounds belonging to flavonoids, are abundant in red, blue, and black berries but also present in red wine and dark-colored vegetables [18,19].

ACs have been associated with many protective biological effects, including anti-oxidative, anti-carcinogenic, antimicrobial, and anti-inflammatory properties [17,20,21]. Due to their phenolic structure, ACs exhibit an anti-oxidative capacity in vivo as they scavenge reactive oxygen species (ROS) [20,22], also a classical effect of 5-ASA [23]. After ingestion, ACs largely bypass absorption in the upper gastrointestinal tract, reaching the colon intact, where they are metabolized by microbiota through deglycosylation and further degraded into vanillic, protocatechuic, *p*-coumaric, gallic, and syringic acids (i.e., phenolic acids) [24]. ACs interrupt the pro-inflammatory signaling and are inhibitors of 5-lipoxygenase, a key enzyme implicated in the arachidonic acid pathway for the biosynthesis of active leukotrienes (mainly via the unstable intermediate LTA_4_ to LTB_4_ and 5-HETE [25,26]). In the presence of flavonoids, monocytes release less tumor necrosis factor (TNF) and interleukin (IL)-8 [27]. ACs also impede the activation of nuclear factor κB (NF-ĸB) by the inhibition of proteasomal function. NF-ĸB, as well as TNF and IL-8, constitutes key molecules orchestrating inflammation, including IBD [17,28,29].

In respect to IBD, recent studies have highlighted the therapeutic potential of ACs in UC by modulating inflammatory pathways. AC are known to restore the intestinal barrier integrity by upregulating tight junction proteins, preventing increased permeability and inflammation. A recent systematic review highlighted that ACs influence gut health by promoting the growth of *Bacteroidetes* while suppressing *Firmicutes*. They also enhance the production of short-chain fatty acids, lower intestinal pH, reduce intestinal permeability, and contribute to the proliferation of goblet cells. Additionally, ACs upregulate tight junction proteins, like occludin, and improve the length and height of intestinal villi [24]. Further scientific evidence supports that ACs also enhance gut microbiota composition, increasing beneficial bacteria such as the *Lactobacillus* and *Bifidobacterium* genera, which promote the production of butyrate, a key anti-inflammatory short-chain fatty acid [24,30]. Cyanidin-3-O-glucoside, a prominent AC active component, has exhibited specific benefits in reducing oxidative stress and inhibiting NF-κB signaling, critical in UC pathogenesis. Additionally, ACs scavenge ROS and inhibit the overexpression of key inflammatory mediators, such as the cytokines IL-1β, IL-6, and IFN-γ, supporting mucosal healing [31]. Despite these encouraging findings, the precise mechanisms of ACs in UC still remain incompletely understood, warranting further research to optimize their clinical application.

Moreover, several research groups [32,33,34,35,36,37,38], including ours [21,39,40,41,42,43,44], reported a beneficial effect of ACs on preclinical models of UC. In view of the aforementioned results, the potential of ACs in a small, uncontrolled pilot trial in 13 patients with UC was tested [45]. For six weeks, patients received a daily AC-rich extract from bilberries (*Vaccinium myrtillus*). Strikingly, clinical disease activity, as well as endoscopic histological and biochemical indicators for intestinal inflammation, markedly improved. Side effects were not observed. These data suggest ACs as a potential adjunctive treatment option in UC with very few, if any, side effects [45,46].

While these findings are encouraging, it is important to stress that preclinical studies are conducted in controlled environments using animal models, which do not fully replicate the complexity of human UC, including its variable disease course, immune responses, and the influence of factors like diet and microbiota [47]. Similarly, clinical studies to date, including our pilot trials, have been limited by small sample sizes, single-center designs, or lack of randomization and control groups, which restrict the generalizability and reproducibility of their findings. Such limitations are known to confine the conclusions and applicability of clinical trials [48,49].

To overcome the abovementioned limitations, this study utilizes a multi-center, double-blind, placebo-controlled, parallel-group methodology to rigorously assess the efficacy, safety, and tolerability of an anthocyanin-rich extract (ACRE) in a larger and more diverse population of patients with moderately active UC.

## 2. Materials and Methods

### 2.1. Study Population

Patients with moderately or severely active UC were recruited between April 2019 and March 2021 at six IBD centers in Switzerland. Moderately or severely active UC was defined as a Mayo score of 6–12 with an endoscopic sub-score ≥ 2. Patients aged 18–70 years and diagnosed with UC for at least three months with the disease extending at least 15 cm from the anal verge were included. Current oral or rectal 5-ASA/sulfapyridine (SP) use or a history of oral or rectal 5-ASA/SP was allowed. Furthermore, patients were eligible for the study if they fulfilled one of the following criteria: a. Steroid intake up to 30 mg/day as well as a history of steroid dependency, refractory, or intolerance, including no steroid treatment due to earlier side effects. OR b. Active disease despite induction therapy with 5-ASA agents, either mesalamine (2–4.8 g/day) or sulfasalazine (4–6 g/day), administered for at least two weeks. Topical treatment with 5-ASA was permissible but not sufficient for inclusion in the study. OR c. Intolerance to oral 5-ASA or azathioprine. OR d. Active disease despite thiopurine (adequately dosed according to treatment guidelines [50], such as 2–3 mg/kg for azathioprine) or methotrexate treatment administered for at least 12 weeks. OR e. Active disease despite treatment with biologics effective in UC or calcineurin inhibitors. No restrictions regarding other IBD therapies applied the following: Azathioprine/6-mercaptopurine was allowed, providing that the dose had been stable for 8 weeks prior to baseline and had been initiated at least two months before screening. TNF inhibitors (i.e., infliximab, adalimumab, or golimumab) were allowed, providing that the dose was unchanged for at least two months prior to baseline and during the study treatment period. Vedolizumab and tofacitinib were allowed, providing that the dose remained unchanged for at least two months prior to baseline and during the study treatment period.

The exclusion criteria were a suspicion or diagnosis of Crohn’s disease, ischemic colitis, radiation colitis, indeterminate colitis, infectious colitis, diverticular disease associated colitis, microscopic colitis, massive pseudopolyposis, or a colonic stenosis that could not be passed endoscopically, acute severe UC (as defined by Truelove and Witt’s criteria [51]), and/or signs of systemic toxicity, UC limited to the rectum (disease which extends < 15 cm above the anal verge), long term treatment with antibiotics or non-steroidal anti-inflammatory drugs (NSAID) within two weeks prior to screening (one short treatment regime for antibiotics and occasional use of NSAID was allowed) and within at least 30 days after the last treatment of the experimental product prior to enrolment history of malignancy, a history or presence of any clinically significant disorder that, in the opinion of the investigator, could impact on the patient’s ability to adhere to the study protocol and study procedures or would confound the study result or compromise patient safety.

All patients signed an informed consent form. The study was approved by the ethics committee of each center (BASEC2017-00156, approval date: 14 February 2019) and was conducted in accordance with the latest revision of the Declaration of Helsinki [52] as well as the guidelines of Good Clinical Practice [53]. Moreover, the study was registered in the US online database of clinical research studies ClinicalTrials.gov (NCT04000139). The reporting of this trial followed the CONSORT guidelines for randomized controlled trials [54]. All authors were offered full access to the study data and reviewed and approved the final manuscript.

### 2.2. Study Design and Procedures

This was a multi-center, randomized, double-blind, placebo-controlled trial. Patients with moderate-to-severe active UC, despite state-of-the-art 5-ASA, steroids, and immunosuppressive biological treatment, were enrolled. Patients were randomized 2:1 (ACRE: placebo).

Upon study entry, all patients were instructed to continue their current medications unchanged. They either received a standardized ACRE (referring to 800–1000 mg AC) administered per os three times daily or an optically identical placebo. Of note, similar or even lower AC doses have been previously administered in human studies investigating (extra)intestinal beneficial actions with favorable results [45,55,56]. The administered ACRE was a standardized ethanolic bilberry extract, provided by Walther Riemer GmbH (Nimbo Green, Ningbo, China). The standardized ACRE cyanidin-3-O-glucoside was its most prominent component (at least 36%; also kindly refer to Supplementary Materials), as determined by means of high-performance liquid chromatography. Additional active compounds included cyanidin-3-O-galactoside, cyanidin-3-O-arabinoside, delphinidin-3-O-galactoside, delphinidin-3-O-glucoside, delphinidin-3-O-arabinoside, petunidin-3-O-galactoside, petunidin-3-O-glucoside, petunidin-3-O-arabinoside, peonidin-3-O-glucoside, peonidin-3-O-galactoside, peonidin-3-O-arabinoside, malvidin-3-O-galactoside, malvidin-3-O-glucoside, and malvidin-3-O-arabinoside.

As the ACRE capsule preparation contained purple compounds (which could not be eliminated during the production process), we added equally purple powder to the placebo capsule preparation in order to avoid any unblinding. The total duration of the investigational product administration amounted to eight weeks (56 days). All participating physicians were blinded to the treatment assignment throughout the study. After enrollment, patients underwent a physical examination and laboratory blood tests, including a complete blood count, liver function tests, and C-reactive protein, which were performed at both baseline and at the end of the phase protocol. Patients also underwent flexible rectosigmoidoscopy at study entry (baseline) and at week 8, and endoscopic activity was determined according to the endoscopic Mayo index sub-score [57] by a local and a central reader.

### 2.3. Clinical Assessment and Trial Endpoints

The primary objective of the study was to evaluate the efficacy of the ACRE preparation in subjects with moderate-to-severe active UC according to clinical, endoscopic, histological, and biochemical markers. Secondary objectives included the effects of ACRE on quality of life (QoL) as well as its safety.

(Mayo score of 6–12, endoscopic sub-score ≥ 2) by comparing the clinical response rate of subjects on an ACRE versus a placebo arm at week 8. As clinical response was defined as a reduction in the total Mayo score (TMS) ≥ 3 points. Of note, the mentioned cut-off for clinical response exhibits an established validity in several studies previously [58,59,60]. The secondary objectives included the evaluation of (1) the ACRE efficacy at week 8, where clinical remission is defined as a Mayo score ≤ 2, with no individual sub-score > 1; (2) ACRE safety and tolerability; (3) the efficacy of an ACRE preparation in subjects with moderately active UC compared to placebo in clinical remission, clinical response, and clinical symptoms; (4) ACRE efficacy in subjects with moderate-to-severe active UC compared to the placebo in endoscopic and histological remission and response; and (5) quality of life (QoL) in patients of the ACRE arm.

The primary endpoint was the clinical response at week 8, with clinical response defined as a reduction in TMS by ≥3 points, similar to previous studies [58,59,60].

Secondary endpoints were as follows:

- The proportion of patients achieving symptomatic remission at week 8, defined as both a Mayo rectal bleeding sub-score of 0 and a stool frequency sub-score of 0 or 1 (with at least 1 point decrease from baseline, week 0) (patient-reported outcome) [PRO2] [61].
- The proportion of patients without rectal bleeding at week 8, defined by a Mayo rectal sub-score bleeding of 0.
- The proportion of patients with normal or enhanced stool frequency at week 8, defined by a Mayo stool frequency sub-score of 0 or 1 (with at least 1 point decrease from baseline, week 0).

Other secondary endpoints assessed rectal bleeding, clinical response, and remission at week 4; durable remission (i.e., remission in weeks 8 and 12); and clinical response at week 8, according to the modified Mayo score, defined as a three-point and ≥30% drop from baseline of the sum of the rectal bleeding, stool frequency, endoscopy score (excluding friability), and physicians’ global assessment (PGA).

- The proportion of patients with endoscopic remission at week 8, defined by a modified Mayo endoscopic sub-score [57] of 0 or 1 (excluding friability).
- The proportion of patients with histological remission at week 8, defined by a Geboes index [62] of grade 0 or 1.
- Mean change in fecal calprotectin (FC) concentrations at weeks 1, 2, 4, and 8 compared to baseline, week 0.
- Mean change in steroid dosage compared to baseline for patients in remission at week 8 to 12.
- Mean change in each of the short inflammatory bowel disease questionnaire (SIBDQ) [63] sub-domains at week 8 compared to baseline (week 0).

The rate and time point of premature study withdrawals between both arms were also explored in the data analysis.

Data acquisition and management were performed using OpenClinica Community Edition, version 3.14, and OpenClinica eCRF.

### 2.4. Analysis Plan

A sample size of 112 patients was initially planned, leading to 100 patients completing the study, assuming a drop-out rate of 12%, based on a frequentist power calculation. According to our previous data, a clinical response was assumed in the active treatment arm of 55% and in the placebo of 25%. For a 1:1 placebo vs. verum randomization ratio, a patient number of 41 per group would be sufficient to achieve statistical significance with a power of 80%, assuming an α-error of maximal 5%. For the planned 1:2 ratio, the respective numbers were 33 (placebo) and 66 (verum) patients for both groups. The analysis of the primary endpoint was conducted on the FAS (full analysis set) and PPS (per protocol set). A data analysis was performed using SAS, version 9.4 (Windows x64 version). Missing singular data items were carried forward. This did not apply, of course, when entire visits were missed (p.ex., in the case of premature study termination).

## 3. Results

### 3.1. Study Population

**Figure 1:**
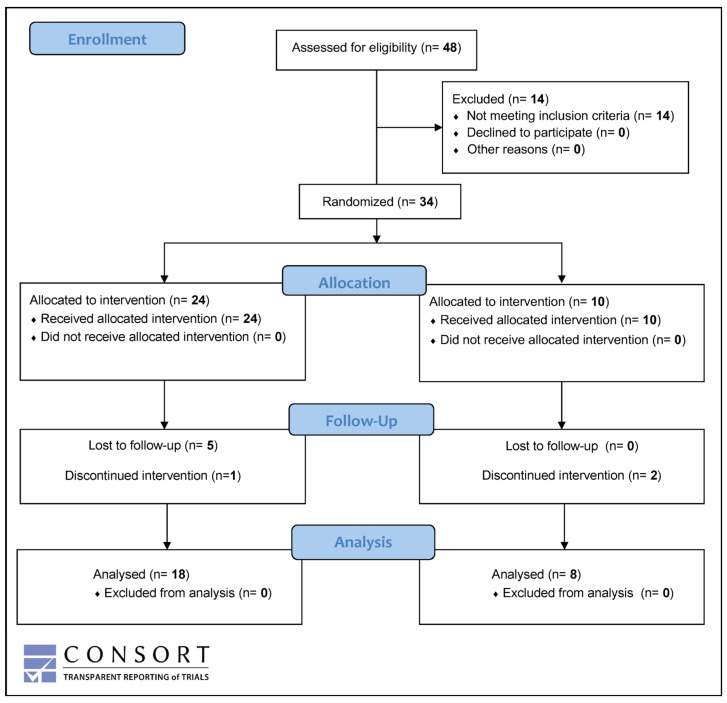
CONSORT flow diagram with study design and participants progression. It details the number of patients screened, randomized, and allocated to treatment groups and those included in the final analysis.

Out of 48 patients screened in a total of six Swiss trial centers, 34 (70.9%) were enrolled and randomized, whereas 14 (29.1%; 4 females and 10 males) were considered screening failures. A detailed flowchart outlining the patient recruitment process, following the CONSORT guidelines [54], is provided in Figure 1.

Five randomized ACRE and two placebo patients prematurely terminated the study before the scheduled end of treatment at visit 3 (week 8). One ACRE patient started prednisone (20 mg/day) treatment at visit 1 and was excluded from all analyses for violation of the protocol. Thus, 18 ACRE and 8 placebo patients could be analyzed in the per protocol set (PPS).

At screening, the majority of patients (69.2%) were on concomitant steroid medication. A total of 16.7% in the bilberry group and 25% in the placebo group received anti-TNF treatment. Patient baseline characteristics are enclosed in Table 1.

### 3.2. Efficacy Analysis

#### 3.2.1. Primary Endpoint (Clinical Response at Week 8)

Clinical response at week 8 was achieved in 9 out of 18 ACRE patients and 3 out of 8 (3/8) placebo patients (Table 2). This corresponds to an odds ratio (OR) for the response of 1.667 in favor of the ACRE arm, with a 90% confidence interval (CI) between 0.399 and 6.963 and a one-sided *p* = 0.278 in the logistic analysis, and the primary endpoint was not met.

**Figure 2:**
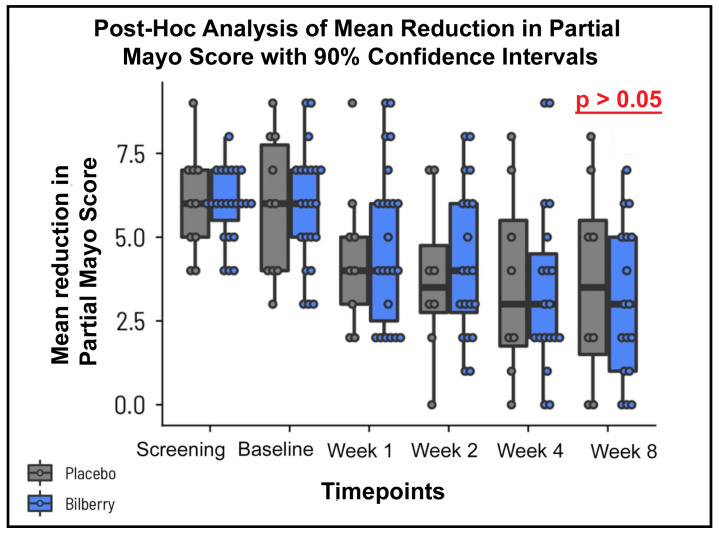
Illustration of the mean reduction in the partial Mayo score from baseline to week 8 for participants treated with ACRE (anthocyanin-rich extract) and placebo. Error bars represent the 90% confidence intervals for the mean reduction. Although the results indicate a trend toward amelioration in the ACRE group, the differences remain statistically non-significant.

In a post-hoc analysis, the mean reduction in the partial Mayo score was 2.61 ± 2.79 for the ACRE arm and 2.00 ± 3.07 for the placebo arm (Figure 2, not significant). Assuming a normal distribution, the 90% CI of the change in TMS does not include the point 0 (no change) for ACRE, while this point is within the 90% CI for the placebo arm.

An interesting finding of this study is the unusually high percentage of placebo response, i.e., 37.5% for the clinical response and 62.5% for the Mayo score amelioration.

#### 3.2.2. Secondary Endpoints

The secondary endpoint of clinical remission at week 8 was achieved in 9/18 patients of the ACRE arm versus 3/8 patients of the placebo arm (CI 0.399–6.963; *p* = 0.278, not significant), even though we noted numerical improvements in both the individual sub-scores for rectal bleeding and stool frequency (Table 3).

### 3.3. Endoscopic Remission at Week 8

The endoscopic findings (with central reading and site reading) yielded similar results, which are inconclusive and fail to demonstrate therapeutic effects.

#### Change in Fecal Calprotectin Endpoint vs. Baseline

**Figure 3:**
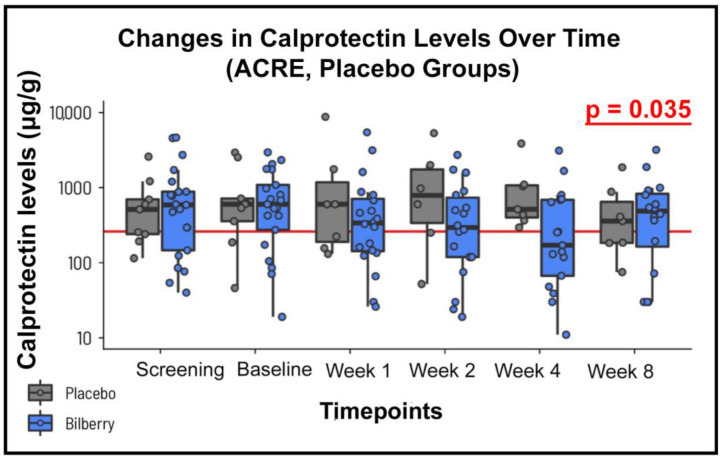
Illustration of fecal calprotectin levels (μg/g) across the observation period in patients treated with ACRE (anthocyanin-rich extract) and placebo. Measurements were taken at screening, baseline, week 4, and week 8. A statistically significant reduction in calprotectin levels was observed in the ACRE group compared to the placebo (*p* = 0.035), suggesting potential anti-inflammatory effects of ACRE.

FC remained practically the same in the placebo group (Figure 3) between baseline and end-of-experiment values (947 ± 1039 to 1040 ± 1179), whereas it significantly decreased for the ACRE group (1049 ± 1139 to 557 ± 756 μg/g, *p* = 0.035). No difference for any of the other secondary endpoints was noted.

### 3.4. Safety Analysis

In our safety analysis, adverse events (AEs) were recorded and centrally reviewed by the trial’s safety monitoring board. Overall, in a total of 29 patients, AEs were reported by the study conductors. The most commonly reported AEs included stool discoloration or headaches. Importantly, no serious adverse events (SAEs) directly attributed to the intervention were reported during the study. Among the treated patients, a total of 68 adverse events, 2 of which were regarded to be serious, were recorded in 29 patients. Specifically, one patient developed infectious colitis before enrollment, and another experienced an exacerbation of UC requiring hospitalization. Both events were determined to be unrelated to the intervention.

While the treatment group exhibited a higher frequency of AEs compared to the placebo group (43 for the ACRE arm vs. 25 for placebo, which can be expected in view of the fact that stool discoloration represented the most frequent AE), this difference did not reach statistical significance. Most adverse events, such as nausea or transient fatigue, were mild and resolved spontaneously without requiring medical intervention or discontinuation of treatment. Importantly, no patterns indicative of systemic toxicity or unexpected adverse effects were observed during the study.

These findings suggest that the intervention is generally well tolerated, with an acceptable safety profile consistent with prior studies in similar populations. This observation aligns with established data for similar interventions, further reinforcing the absence of new safety concerns.

Overall, ACRE was very well tolerated, and no new safety signals were detected, underscoring its potential as a safe therapeutic option in this context.

## 4. Discussion

In this multi-center, randomized, double-blind, placebo-controlled, parallel-group, phase IIa study, we aimed to evaluate the efficacy, safety, and tolerability of ACRE in patients with UC. Although the clinical response at the end of the study did not differ between the two arms, a statistically significant difference was recorded in the secondary endpoint of FC.

FC serves as a valuable indicator of inflammation within the intestines [64,65] and has become an integral component of routine testing for diagnosing and monitoring IBD. According to a recent meta-analysis, FC is an inexpensive, valuable, and rather accurate predictor of IBD relapses [64]. Additionally, further evidence has established FC as a useful marker recognizing an early response to IBD treatment since it correlates robustly with serologic markers, endoscopic inflammation, and disease activity indices of IBD subjects [65].

As aforementioned, our research group firstly reported in 2013 in a pilot study [45] the beneficial effects of bilberries on UC patients, with results that we could also confirm later on [40]. Comparable beneficial results have been demonstrated independently by Kropat et al. [66]. An ongoing relevant Australian clinical trial with a double-blind, randomized, controlled, multi-arm design has been recently announced [67], investigating the effect of the administration of ACs and/or multi-strain probiotics on UC patients.

In respect to the available in vivo preclinical scientific evidence, we reported as early as 2011 that bilberries and their AC positively impacted an experimental acute and chronic colitis mouse model [21]. Later, and again by utilizing the same murine model mimicking human UC and UC-associated cancer, we demonstrated [41] that AC ingestion could avert the onset and progression of murine tumors in the colon. Comparable favorable results of ACs on IBD murine models have been henceforth published by other study groups [34,35,36,68,69].

Interestingly, there is emerging evidence that AC administration offers beneficial effects beyond IBD to further intestinal diseases such as colorectal carcinoma and irritable bowel syndrome [70]. Bilberries and/or ACs have also been investigated as a treatment targeting extraintestinal organs in various settings and have shown a positive impact in clinical trials, among others, for vascular health and cognitive function [55] or metabolic syndrome and associated conditions [29]. A very recent US study with a large sample of patients (n = 37,232) found a direct inverse relationship between overall mortality risk and consumption of diverse berries and their containing flavonoids [19].

The health-promoting effects of AC are attributed to their antioxidant properties, enabled by their phenolic structure, which neutralizes ROS [44]. Additionally, ACs display antimicrobial effects, such as against *Bacillus cereus* and *Helicobacter pylori* [71]. They also reduce the expression of various genes involved in atherosclerosis formation in animal models [72]. Moreover, ACs inhibit inflammation-promoting pathways in immune cells [44].

As stressed in the result section, an unusually high percentage of placebo response for the Mayo score improvement was noted. In this respect, a Cochrane meta-analysis [73] of 61 studies estimated the placebo response and remission rates of induction treatment for UC (adult population) to be 33% and 12%, respectively. The placebo effect and rates of remission fluctuated based on several factors, including the severity of endoscopic disease and the rectal bleeding score upon trial initiation, the type of medication used, the duration of the disease, and the specific time when the primary outcome was assessed [73].

In a second more recent systematic review with meta-analysis (2022), clinical, endoscopic, histological, and safety placebo rates in the induction and maintenance trials of UC have been evaluated [74]. After considering a total number of 119 trials (of which 92 in induction and 27 in maintenance), the abovementioned parameters for the induction studies were 11%, 19%, and 15%, respectively. Again, the authors deduced that placebo response rates observed in trials for UC differ depending on the endpoint evaluated, whether it pertains to response assessment or achieving remission, and whether the trial focuses on induction or maintenance.

The purple color of the drug as well as the placebo might be partially responsible for the high placebo rates. As a possible interpretation of this unexpected placebo effect, studies conducted in the past have identified the color of the placebo pills to correlate with outcomes [75,76,77]. Thus, the colors of the drugs may impact how their effects are perceived and may also play a role in their effectiveness. Additionally, there appears to be a connection between the coloring of the drugs that affect the central nervous system and the conditions they are prescribed for. In this context, there is evidence that red, yellow, and orange colors of pills are linked to a stimulant effect, whereas blue, purple, and green are connected to a tranquillizing sedative effect [75,76,77].

The strengths of this study include the double-blinded, randomized, and multi-center study design. The low number of participants is the main limitation of this study; this is at least partially due to restrictions during the COVID-19 pandemic, since individual patients, as aforementioned, impacted the outcome relevantly. However, this did not prevent a statistical significance for FC between the two study arms. A further limitation of this study is the lack of dietary intake assessment. Without tools such as a 24-h dietary recall or a dietary questionnaire, it is difficult to rule out the possibility that the participants independently adjusted their consumption of AC-rich foods during the study period, influenced by their awareness of the health benefits of such compounds. Future studies should include dietary monitoring to better control for this potential confounder.

## 5. Conclusions

In conclusion, ACRE did not achieve a clinical response at 8 weeks in patients with moderate-to-severe UC. However, despite recruitment challenges related to COVID-19 that constrained the sample size and some analyses, significant reductions in FC levels were observed with the ACRE treatment. FC is a recognized biomarker of intestinal inflammation, and its reduction is associated with improvements in mucosal healing and disease control in UC, highlighting a potential therapeutic effect that warrants, albeit, further investigation.

## Figures and Tables

**Table 1:** Baseline characteristics.

	ACRE	Placebo	All Patients
Gender (f/m) All randomized patients	10/14	5/5	15/19
Gender (f/m) Per protocol set	8/10	4/4	12/14
Total Mayo Score (Mean value ± SD) at Screening	8.43 ± 1.31	8.50 ± 1.90	
Steroids/no steroids	14 (77.8%) 4 (22.2%)	4 (50.0%) 4 (50.0%)	18 (69.2%) 8 (30.8%)
TNF tx/no TNF tx	3 (16.7%) 15 (83.3%)	2 (25.0%) 6 (75.0%)	5 (19.2%) 21 (80.8%)

ACRE, anthocyanin-rich extract; f/m, female/male; SD, standard deviation; TNF, tumor necrosis factor; tx, treatment.

**Table 2:** Categorized change in the total Mayo score.

Change in Total Mayo Score	Treatment			
	ACRE		Placebo	
	Patients	%	Patients	%
Clinical response	9	50.0%	3	37.5
Improved	5	27.8%	2	25.0
Unchanged	2	11.1%	2	25.0
Worsened	2	11.1%	1	12.5
All	18	100.0%	8	100.0

ACRE, anthocyanin-rich extract.

**Table 3:** Categorized changes at week 8.

	Treatment				*p*-Value
	ACRE		Placebo		
	Patients	%	Patients	%	
Symptomatic remission	8/18	44.4	3/8	37.5	0.371
No bleeding	13/18	72.2	4/8	50.0	0.139
Normal stool frequency	10/18	11.1	4/8	50.0	0.397
Endoscopic remission	4/18	22.2	2/8	25	0.412

ACRE, anthocyanin-rich extract.

## Data Availability

Data are available on request due to privacy/ethical restrictions.

## Supplementary Materials

The following supporting information can be downloaded at https://www.mdpi.com/article/10.3390/nu16234197/s1, Bilbrerries specification.
