# Supplementary material for: Efficacy and Safety of Anthocyanin-Rich Extract in Patients with Ulcerative Colitis: A Randomized Controlled Trial
Source: Nutrients. 2024 Dec 4;16(23):4197. doi: 10.3390/nu16234197 (PMC11644667; doi:10.3390/nu16234197)
Supplement: Supplementary file 1 [file nutrients-16-04197-s001.zip › nutrients-3327370-supplementary.pdf]

WALTER J. RIEMER GmbH
Hermannstr. 40
20095 Hamburg
Tel: +49 40 33 73 55
Fax: +49 40 33 73 15
Email: info@wj-riemer.de

Specification

**Product Name**		**BILBERRY 36%**		
**Product Number**		**2050**		
**Date**	**October 22, 2015**		**Page**	**1 / 2**

Characteristics	Value	Lower limit	Upper limit
**Color**			
**Visual evaluation in sample vial**	**violet to dark violet**		
**Appearance/condition**			
**Visual evaluation in sample vial**	**powder**		
**Loss on drying**			
**1 g sample, 110 degr. C, 3 hours**	**%**	**max.**	**4,5**
**Particle size**			
**Sieve Analysis, 10 min**			
**< 0.600 mm (USS# 30)**	**%**	**100,0**	**min.**
**Heavy metals**			
**Heavy metals calculated as lead**	**ppm**	**max.**	**30**
**GLC, Headspace**			
**GLC Headspace**			
**Ethanol**	**ppm**	**max.**	**1000**
**Methanol**	**ppm**	**max.**	**10**
**Identity (TLC)**			
**thin layer chromatography**	**comparable to standard**		
**Assay (Pharmacopoeia)**			
**Quantitative determ.**			
**Anthocyanins (HPLC)**			
**calculated as**			
**Cyanidin-3-O-glucoside chloride**	**%**	**36,0**	**min.**
**Quantitat. determ.**			
**Anthocyanidins (HPLC)**			
**calculated as Cyanidin chloride**	**%**	**max.**	**1,0**

WALTER J. RIEMER GmbH
Hermannstr. 40
20095 Hamburg
Tel: +49 40 33 73 55
Fax: +49 40 33 73 15
Email: info@wj-riemer.de

Specification

**Product Name**		**BILBERRY 36%**	
**Product Number**		**2050**	
**Date**	**October 22, 2015**	**Page**	**2 / 2**

Characteristics	Value	Lower limit	Upper limit
**Sulphated ash (Pharmacopoeia)**			
**Sulphated ash (EUR. PH.)**	**%**	**max.**	**3,0**
**Assay**			
**UV spectrum in methanolic HCl 0,1%**			
**Anthocyanidin, as Delphinidin**	**%**	**25,0**	**min.**
**Total aerobic count**			
**Poured Plate**	**per g**	**max.**	**1000**
**Moulds and yeasts**			
**Poured Plate**	**per g**	**max.**	**100**

Specification processed automatically and therefore has no signature.
